# Fractional Hermite–Hadamard inequalities for $(s,m)$-convex or *s*-concave functions

**DOI:** 10.1186/s13660-018-1829-1

**Published:** 2018-09-12

**Authors:** Tieyan Lian, Wei Tang, Rui Zhou

**Affiliations:** 1 College of Light Industry Science and Engineering, Shaanxi University of Science and Technology, Xi’an, China; 2 College of Electrical and Information Engineering, Shaanxi University of Science and Technology, Xi’an, China; 3 College of Arts and Sciences, Shaanxi University of Science and Technology, Xi’an, China

**Keywords:** Hermite–Hadamard inequality, $(s,m)$-convex function, Riemann–Liouville fractional integrals

## Abstract

In this article, fractional integral is considered. Some new upper bounds of the distance between the middle and left of Hermite–Hadamard type inequalities for fractional integrals are established for $(s,m)$-convex or *s*-concave functions.

## Introduction

Let **R** be the set of real numbers, $I \subset\mathbf{R}$, $I^{o}$ be the interior of *I*. The following is the definition of convex functions which is well-known knowledge in mathematical literature: $f:I \rightarrow\mathbf{R}$ is said to be convex if the inequality

1.1 $$ f\bigl(\lambda x+(1-\lambda)y\bigr)\leq\lambda f(x)+(1-\lambda)f(y) $$

holds for all $x,y\in I$ and $\lambda\in[0,1]$. We say that *f* is concave if −*f* is convex.

For a convex function, many equalities or inequalities have been established, but Hermite–Hadamard’s integral inequality is one of the most important ones, which is stated as follows [1]:

If $f:I \rightarrow\mathbf{R}$ is a convex function on *I* and $a,b\in I$ with $a< b$, then

1.2 $$ f \biggl( \frac{a+b}{2} \biggr) \leq\frac{1}{b-a} \int^{b}_{a} f(x)\,dx \leq\frac{f(a)+f(b)}{2} $$

holds. Both the inequalities hold in the reversed direction if *f* is concave.

Inequality (1.2) has been a subject of extensive research since its discovery, and a number of papers have been written providing noteworthy extensions, generalizations, and refinements for some new class of convex functions.

For example, Orlicz [2] introduced the definition for *s*-convexity of real-valued mappings.

In [3], Hudzik and Maligranda showed the class of mappings which are *s*-convex in the second sense in the following way: A mapping $f:I\subseteq\mathbf{R}^{+}=[0,\infty)\rightarrow\mathbf{R}$ is said to be *s*-convex in the second sense on *I* if the inequality

1.3 $$ f\bigl(tx+(1-t)y\bigr)\leq t^{s}f(x)+(1-t)^{s}f(y) $$

holds for all $x,y\in I$ and for some fixed $s\in(0,1]$.

Inequality (1.3) holds in the reversed direction if *f* is *s*-concave in the second sense.

In [4], Dragomir and Fitzpatrick proved a variant of Hadamard’s inequality for functions which are *s*-convex in the second sense.

### Theorem 1.1

([4])

*Suppose that* $f:I\subseteq \mathbf{R}^{+}\rightarrow\mathbf{R}$ *is* *s*-*convex in the second sense on* *I*, *where* $s\in(0,1]$, *and let* $a,b\in I$ *with* $a< b$. *If* *f* *is in* $L([a,b])$, *then the following double inequality holds*:

1.4 $$ 2^{s-1}f\biggl(\frac{a+b}{2}\biggr)\leq\frac{1}{b-a} \int_{a}^{b}f(x)\,dx\leq \frac{f(a)+f(b)}{s+1}. $$

*Both inequalities* (1.4) *hold in the reversed direction if* *f* *is* *s*-*concave in the second sense*.

In a recent paper, Eftekhari [5] defined the class of $(s,m)$-convex functions in the second sense as follows: A mapping $f:I\subseteq\mathbf{R}^{+}=[0,\infty)\rightarrow\mathbf{R}$ is said to be $(s,m)$-convex in the second sense on *I* if the inequality

$$f\bigl(tx+m(1-t)y\bigr)\leq t^{s}f(x)+m(1-t)^{s}f(y) $$

holds for all $x,y\in I$ and for some fixed $(s,m)\in(0,1]^{2}$.

It is easy to observe that for $m=1$, the class of $(s,m)$-convex in the second sense is merely the class of *s*-convex mappings in the second sense defined on $[0,\infty)$.

Our results generalize the following results which are connected with the left-hand side of (1.2).

### Lemma 1.1

([4])

*Let* *f* *be defined on* *I* *and differentiable on* $I^{o}$ *such that* $a,b\in I$ *with* $a< b$, $f'\in L[a,b]$, *then the following equality holds*:

1.5 $$ \begin{aligned}[b] &f \biggl( \frac{a+b}{2} \biggr) - \frac{1}{b-a} \int_{a}^{b}f(t)\,dt \\ &\quad =\frac{b-a}{4} \biggl[ \int_{0}^{1}tf' \biggl( t \frac{a+b}{2}+(1-t)a \biggr) \,dt+ \int_{0}^{1}(t-1)f' \biggl( t \frac{a+b}{2}+(1-t)b \biggr) \,dt \biggr] . \end{aligned} $$

### Theorem 1.2

([5])

*Let* *f* *be defined on* *I* *and differentiable on* $I^{o}$ *such that* $a,b\in I$ *with* $a< b$, $f'\in L[a,b]$. *If* $\vert f'\vert $ *is* $(s,m)$-*convex in the second sense on* $[a,b]$ *for* $(s,m)\in(0,1]^{2}$, *then the following inequality holds*:

1.6 $$ \begin{aligned}[b] & \biggl\vert f \biggl( \frac{a+b}{2} \biggr) -\frac{1}{b-a} \int _{a}^{b}f(t)\,dt \biggr\vert \\ &\quad \leq \frac{b-a}{4(s+2)} \biggl[ 2 \biggl\vert f' \biggl( \frac{a+b}{2} \biggr) \biggr\vert + \frac{m}{s+1} \biggl( \biggl\vert f'\biggl(\frac{a}{m}\biggr) \biggr\vert + \biggl\vert f'\biggl(\frac {b}{m}\biggr) \biggr\vert \biggr) \biggr] . \end{aligned} $$

In [6], in terms of $(s,m)$-convex functions in the second sense, Sarikaya et al. established Hermite–Hadamard’s inequalities for Riemann–Liouville fractional integral. Furthermore, some results connected with the right-hand side of (1.2) were obtained for Riemann–Liouville fractional integral.

Different from [6], our aim of this work is to establish some upper bounds to the left-hand side of (1.2) for Riemann–Liouville fractional integral. When we take appropriate parameters, we can get (1.5) and (1.6).

To get our main results, we will give some necessary definitions and mathematical preliminaries of fractional calculus theory which are used further in this paper. For more details, one can consult [7–9].

### Definition 1.1

([7])

Let $f\in L_{1}[a,b]$. The Riemann–Liouville integrals $J_{a^{+}}^{\alpha}f$ and $J_{b^{-}} ^{\alpha}f$ of order $\alpha>0$ with $a\geq0$ are defined by

$$\begin{aligned}& J_{a^{+}}^{\alpha}f(x)=\frac{1}{\Gamma(\alpha)} \int_{a}^{x}(x-t)^{ \alpha-1}f(t)\,dt,\quad x>a, \\ & J_{b^{-}}^{\alpha}f(x)=\frac{1}{\Gamma(\alpha)} \int_{x}^{b}(t-x)^{ \alpha-1}f(t)\,dt,\quad x< b, \end{aligned}$$

respectively. Here, $\Gamma(\alpha)$ is the gamma function and $J_{a^{+}}^{0}f(x)=J_{b^{-}}^{0 }f(x)=f(x)$.

## Main results

For establishing some new fractional integral inequalities of Hermite–Hadamard type for *m*-convex or $(s,m)$-convex functions, we need the fractional integral identity below.

### Lemma 2.1

*Let* *f* *be defined on* *I* *and differentiable on* $I^{o}$ *such that* $a,b\in I$ *with* $a< b$, $f'\in L[a,b]$, *then the following equality holds*:

2.1 $$ \begin{aligned}[b] & f\biggl(\frac{a+b}{2}\biggr)+ \frac{\Gamma(\alpha+1)}{b-a} \biggl[ \biggl(\frac{2}{a-b}\biggr)^{ \alpha-1}J_{{(\frac{a+b}{2})}^{+}}^{\alpha}f(a) -\biggl(\frac{2}{b-a}\biggr)^{ \alpha-1}J_{{(\frac{a+b}{2})}^{+}}^{\alpha}f(b) \biggr] \\ &\quad =\frac{b-a}{4} \biggl[ \int_{0}^{1}t^{\alpha}f'\biggl(t{ \frac{a+b}{2}}+(1-t)a\biggr)\,dt- \int_{0}^{1}t^{\alpha}f'\biggl(t{ \frac{a+b}{2}}+(1-t)b\biggr)\,dt \biggr] . \end{aligned} $$

### Proof

First of all, let

2.2 $$\begin{aligned}& \frac{b-a}{4} \biggl[ \int_{0}^{1}t^{\alpha}f'\biggl(t{ \frac{a+b}{2}}+(1-t)a\biggr)\,dt- \int_{0}^{1}t^{\alpha}f'\biggl(t{ \frac{a+b}{2}}+(1-t)b\biggr)\,dt \biggr] \\ & \quad = \frac{b-a}{4}(I_{1}-I_{2}). \end{aligned}$$

By integration by parts and making use of the substitution $u=t{\frac{a+b}{2}}+(1-t)a$, we have

2.3 $$\begin{aligned} I_{1}&= \int_{0}^{1}t^{\alpha}f'\biggl(t{ \frac{a+b}{2}}+(1-t)a\biggr)\,dt \\ & =\biggl(\frac{2}{b-a}\biggr)^{\alpha+1} \int_{a}^{\frac{a+b}{2}}(u-a)^{\alpha-1}f'(u)\,du \\ & =\biggl(\frac{2}{b-a}\biggr)^{\alpha+1}\biggl[\biggl( \frac{b-a}{2}\biggr)^{\alpha}f\biggl(\frac{a+b}{2}\biggr)- \alpha \int_{a}^{\frac{a+b}{2}}(u-a)^{\alpha-1}f(u)\,du\biggr] \\ & =\frac{2}{b-a}f\biggl(\frac{a+b}{2}\biggr)+\alpha\biggl( \frac{2}{a-b}\biggr)^{\alpha+1} \int_{\frac{a+b}{2}}^{a}(a-u)^{\alpha-1}f(u)\,du \\ & =\frac{2}{b-a}f\biggl(\frac{a+b}{2}\biggr)+\Gamma(\alpha+1) \biggl( \frac{2}{a-b}\biggr)^{ \alpha+1}J_{(\frac{a+b}{2})^{+}}^{\alpha}f(a). \end{aligned}$$

Analogously, by using the partial integral method and variable substitution, the following equality holds:

2.4 $$ \begin{aligned}[b] & I_{2}= \int_{0}^{1}t^{\alpha}f'\biggl(t{ \frac{a+b}{2}}+(1-t)b\biggr)\,dt \\ &\quad =-\frac{2}{b-a}f\biggl(\frac{a+b}{2}\biggr)+\Gamma(\alpha+1) \biggl(\frac{2}{b-a}\biggr)^{ \alpha+1}J_{(\frac{a+b}{2})^{+}}^{\alpha}f(b). \end{aligned} $$

Thus, using (2.3) and (2.4) in (2.2), we get the required inequality (2.1).

This completes the proof of the lemma. □

### Remark 1

If in Lemma 2.1 we let $\alpha=1$, it is clear that $J_{{(\frac{a+b}{2})}^{+}}^{\alpha}f(a)=\int_{{\frac{a+b}{2}}} ^{a} f(t)\,dt$ and $J_{{(\frac{a+b}{2})}^{+}}^{\alpha}f(b)= \int_{{\frac{a+b}{2}}}^{b} f(t)\,dt$ hold. So in this case, we can see that equality (2.1) is a generalization of equality (1.5) of Lemma 1.1.

It is worth noting that Lemma 2.1 plays a key role in proving our main results.

### Theorem 2.1

*Let* *f* *be defined on* *I* *and differentiable on* $I^{o}$ *such that* $a,b\in I$ *with* $a< b$, $f'\in L[a,b]$. *If* $\vert f'\vert $ *is* $(s,m)$-*convex in the second sense on* $[a,b]$ *for* $(s,m)\in(0,1]^{2}$, *then the following inequality holds*:

2.5 $$ \begin{aligned}[b] & \biggl\vert f\biggl(\frac{a+b}{2}\biggr)+ \frac{\Gamma(\alpha+1)}{b-a}\biggl[\biggl(\frac{2}{a-b}\biggr)^{ \alpha-1}J_{{(\frac{a+b}{2})}^{+}}^{\alpha}f(a)- \biggl(\frac{2}{b-a}\biggr)^{ \alpha-1}J_{{(\frac{a+b}{2})}^{+}}^{\alpha}f(b) \biggr] \biggr\vert \\ &\quad \leq\frac{b-a}{4} \biggl[ \frac{2}{s+\alpha+1} \biggl\vert f'\biggl(\frac{a+b}{2}\biggr) \biggr\vert + mB( \alpha+1,s+1) \biggl( \biggl\vert f'\biggl(\frac{a}{m}\biggr) \biggr\vert + \biggl\vert f'\biggl(\frac{b}{m}\biggr) \biggr\vert \biggr) \biggr] , \end{aligned} $$

*where* $B(x,y)$ *is the classical beta function*, *which may be defined for* $\Re e(x)>0$ *and* $\Re e(y)>0$ *by*

$$B(x,y)= \int_{0}^{1} t^{x-1}(1-t)^{y-1}\,dt. $$

### Proof

By equality (2.1), it follows that

2.6 $$ \begin{aligned}[b] & \biggl\vert f\biggl(\frac{a+b}{2}\biggr)+ \frac{\Gamma(\alpha+1)}{b-a} \biggl[ \biggl( \frac{2}{a-b}\biggr)^{\alpha-1}J_{{(\frac{a+b}{2})}^{+}}^{\alpha}f(a) -\biggl( \frac{2}{b-a}\biggr)^{\alpha-1}J_{{(\frac{a+b}{2})}^{+}}^{\alpha}f(b) \biggr] \biggr\vert \\ &\quad \leq\frac{b-a}{4} \biggl[ \int_{0}^{1}t^{\alpha} \biggl\vert f'\biggl(t{\frac {a+b}{2}}+(1-t)a\biggr) \biggr\vert \,dt \\ &\quad\quad{} + \int_{0}^{1}t^{\alpha} \biggl\vert f'\biggl(t{\frac{a+b}{2}}+(1-t)b\biggr) \biggr\vert \,dt \biggr] . \end{aligned} $$

Since $\vert f'\vert $ is $(s,m)$-convex in the second sense on $[a,b]$, for any $t\in[0,1]$, we obtain

2.7 $$\begin{aligned}& \biggl\vert f'\biggl(t{\frac{a+b}{2}}+(1-t)a\biggr) \biggr\vert \leq t^{s} \biggl\vert f'\biggl( \frac {a+b}{2}\biggr) \biggr\vert +m(1-t)^{s} \biggl\vert f'\biggl( \frac{a}{m}\biggr) \biggr\vert , \end{aligned}$$

2.8 $$\begin{aligned}& \biggl\vert f'\biggl(t{\frac{a+b}{2}}+(1-t)b\biggr) \biggr\vert \leq t^{s} \biggl\vert f'\biggl( \frac {a+b}{2}\biggr) \biggr\vert +m(1-t)^{s} \biggl\vert f'\biggl( \frac{b}{m}\biggr) \biggr\vert . \end{aligned}$$

Using (2.6), (2.7), and (2.8), we have

$$\begin{aligned} & \biggl\vert f\biggl(\frac{a+b}{2}\biggr)+ \frac{\Gamma(\alpha+1)}{b-a} \biggl[ \biggl( \frac{2}{a-b}\biggr)^{\alpha-1}J_{{(\frac{a+b}{2})}^{+}}^{\alpha}f(a)- \biggl( \frac{2}{b-a}\biggr)^{\alpha-1}J_{{(\frac{a+b}{2})}^{+}}^{\alpha}f(b) \biggr] \biggr\vert \\ &\quad \leq\frac{b-a}{4} \biggl[ \int_{0}^{1} 2t^{s+\alpha} \biggl\vert f'\biggl(\frac{a+b}{2}\biggr) \biggr\vert \,dt + \int_{0}^{1}mt^{\alpha}(1-t)^{s} \biggl( \biggl\vert f'\biggl(\frac{a}{m}\biggr) \biggr\vert + \biggl\vert f'\biggl(\frac{b}{m}\biggr) \biggr\vert \biggr) \,dt \biggr] \\ &\quad =\frac{b-a}{4} \biggl[ \frac{2}{s+\alpha+1} \biggl\vert f'\biggl(\frac{a+b}{2}\biggr) \biggr\vert + mB( \alpha+1,s+1) \biggl( \biggl\vert f'\biggl(\frac{a}{m}\biggr) \biggr\vert + \biggl\vert f'\biggl(\frac{b}{m}\biggr) \biggr\vert \biggr) \biggr] . \end{aligned} $$

 □

### Remark 2

If we take $\alpha=1$ in Theorem 2.1, we get inequality (1.6) of Theorem 1.1 which was proved in [5].

### Theorem 2.2

*Let* *f* *be defined on* *I* *and differentiable on* $I^{o}$ *such that* $a,b\in I$ *with* $a< b$, $f'\in L[a,b]$. *If* $\vert f'\vert $ *is* *m*-*convex in the second sense on* $[a,b]$ *for* $m\in(0,1]$, *then the following inequality holds*:

2.9 $$ \begin{aligned}[b] & \biggl\vert f\biggl(\frac{a+b}{2}\biggr)+ \frac{\Gamma(\alpha+1)}{b-a}\biggl[\biggl(\frac{2}{a-b}\biggr)^{ \alpha-1}J_{{(\frac{a+b}{2})}^{+}}^{\alpha}f(a)- \biggl(\frac{2}{b-a}\biggr)^{ \alpha-1}J_{{(\frac{a+b}{2})}^{+}}^{\alpha}f(b) \biggr] \biggr\vert \\ &\quad \leq\frac{b-a}{4} \biggl[ \frac{1}{2(\alpha+2)}\bigl( \bigl\vert f'(a) \bigr\vert + \bigl\vert f'(b) \bigr\vert \bigr) \\ &\quad\quad{} +\frac{m( \alpha+3)}{2(\alpha+1)(\alpha+2)} \biggl( \biggl\vert f'\biggl( \frac{a}{m}\biggr) \biggr\vert + \biggl\vert f'\biggl( \frac{b}{m}\biggr) \biggr\vert \biggr) \biggr] . \end{aligned} $$

### Proof

By equality (2.1), it follows that

2.10 $$\begin{aligned} \begin{aligned}[b] & \biggl\vert f\biggl(\frac{a+b}{2}\biggr)+ \frac{\Gamma(\alpha+1)}{b-a} \biggl[ \biggl( \frac{2}{a-b}\biggr)^{\alpha-1}J_{{(\frac{a+b}{2})}^{+}}^{\alpha}f(a)- \biggl( \frac{2}{b-a}\biggr)^{\alpha-1}J_{{(\frac{a+b}{2})}^{+}}^{\alpha}f(b) \biggr] \biggr\vert \\ &\quad \leq\frac{b-a}{4} \biggl[ \int_{0}^{1}t^{\alpha} \biggl\vert f'\biggl(t{\frac {a+b}{2}}+(1-t)a\biggr) \biggr\vert \,dt+ \int_{0}^{1}t^{\alpha} \biggl\vert f'\biggl(t{\frac{a+b}{2}}+(1-t)b\biggr) \biggr\vert \,dt \biggr] \\ &\quad =\frac{b-a}{4} \biggl[ \int_{0}^{1}t^{\alpha} \biggl\vert f' \biggl( \frac{t}{2}b+\biggl(1- \frac{t}{2}\biggr)a \biggr) \biggr\vert \,dt \\ &\quad\quad{} + \int_{0}^{1}t^{\alpha} \biggl\vert f' \biggl( \frac{t}{2}a+\biggl(1- \frac{t}{2}\biggr)b \biggr) \biggr\vert \,dt \biggr] . \end{aligned} \end{aligned}$$

Since $\vert f'\vert $ is *m*-convex in the second sense on $[a,b]$, for any $t\in[0,1]$, we obtain

2.11 $$\begin{aligned}& \biggl\vert f' \biggl( \frac{t}{2}b+\biggl(1- \frac{t}{2}\biggr)a \biggr) \biggr\vert \leq{\frac{t}{2}} \bigl\vert f'(b) \bigr\vert +m {\biggl(1-\frac{t}{2}\biggr)} \biggl\vert f'\biggl(\frac{a}{m}\biggr) \biggr\vert , \end{aligned}$$

2.12 $$\begin{aligned}& \biggl\vert f' \biggl( \frac{t}{2}a+\biggl(1- \frac{t}{2}\biggr)b \biggr) \biggr\vert \leq{\frac{t}{2}} \bigl\vert f'(a) \bigr\vert +m {\biggl(1-\frac{t}{2}\biggr)} \biggl\vert f'\biggl(\frac{b}{m}\biggr) \biggr\vert . \end{aligned}$$

Using (2.10), (2.11), and (2.12), we have

$$\begin{aligned} & \biggl\vert f\biggl(\frac{a+b}{2}\biggr)+ \frac{\Gamma(\alpha+1)}{b-a} \biggl[ \biggl( \frac{2}{a-b}\biggr)^{\alpha-1}J_{{(\frac{a+b}{2})}^{+}}^{\alpha}f(a)- \biggl( \frac{2}{b-a}\biggr)^{\alpha-1}J_{{(\frac{a+b}{2})}^{+}}^{\alpha}f(b) \biggr] \biggr\vert \\ &\quad \leq\frac{b-a}{4} \biggl[ \int_{0}^{1} \frac{t^{\alpha+1}}{2}\bigl( \bigl\vert f'(a) \bigr\vert + \bigl\vert f'(b) \bigr\vert \bigr)\,dt \\ &\quad\quad {} + \int_{0}^{1} mt^{\alpha}\biggl(1- \frac{t}{2}\biggr) \biggl( \biggl\vert f'\biggl( \frac{a}{m}\biggr) \biggr\vert + \biggl\vert f'\biggl( \frac{b}{m}\biggr) \biggr\vert \biggr) \,dt \biggr] \\ &\quad =\frac{b-a}{4} \biggl[ \frac{1}{2(\alpha+2)}\bigl( \bigl\vert f'(a) \bigr\vert + \bigl\vert f'(b) \bigr\vert \bigr) +\frac{m( \alpha+3)}{2(\alpha+1)(\alpha+2)} \biggl( \biggl\vert f'\biggl( \frac{a}{m}\biggr) \biggr\vert + \biggl\vert f'\biggl( \frac{b}{m}\biggr) \biggr\vert \biggr) \biggr] . \end{aligned}$$

This completes the proof. □

### Corollary 2.1

*Suppose that all the assumptions of Theorem *2.2 *are satisfied*, *and if* $\alpha=1$, *then we have the following inequality*:

2.13 $$ \begin{aligned}[b] & \biggl\vert f \biggl( \frac{a+b}{2} \biggr) -\frac{1}{b-a} \int _{a}^{b}f(t)\,dt \biggr\vert \\ &\leq \frac{b-a}{4} \biggl[ \frac{1}{6}\bigl( \bigl\vert f'(a) \bigr\vert + \bigl\vert f'(b) \bigr\vert \bigr)+ \frac{m}{3}\biggl( \biggl\vert f'\biggl( \frac{a}{m} \biggr) \biggr\vert + \biggl\vert f'\biggl(\frac{b}{m} \biggr) \biggr\vert \biggr) \biggr] .\end{aligned} $$

### Corollary 2.2

*Suppose that all the assumptions of Theorem *2.2 *are satisfied*, *and if* $\alpha=1$, $m=1$, *then inequality* (2.9) *becomes the following inequality which was obtained in* [10]:

$$\biggl\vert f \biggl( \frac{a+b}{2} \biggr) -\frac{1}{b-a} \int _{a}^{b}f(t)\,dt \biggr\vert \leq \frac{b-a}{8} \bigl[ \bigl\vert f'(a) \bigr\vert + \bigl\vert f'(b) \bigr\vert \bigr] . $$

### Theorem 2.3

*Let* *f* *be defined on* *I* *and differentiable on* $I^{o}$ *such that* $a,b\in I$ *with* $a< b$, $f'\in L[a,b]$. *If* $\vert f'\vert ^{q}$ *is* $(s,m)$-*convex in the second sense on* $[a,b]$ *for* $(s,m)\in(0,1]^{2}$ *and if* $q>1$, $q\geq r\geq0$, *then the following inequality holds*:

2.14 $$ \begin{aligned}[b] & \biggl\vert f\biggl(\frac{a+b}{2}\biggr)+ \frac{\Gamma(\alpha+1)}{b-a} \biggl[ \biggl( \frac{2}{a-b}\biggr)^{\alpha-1}J_{{(\frac{a+b}{2})}^{+}}^{\alpha}f(a)- \biggl( \frac{2}{b-a}\biggr)^{\alpha-1}J_{{(\frac{a+b}{2})}^{+}}^{\alpha}f(b) \biggr] \biggr\vert \\ &\quad \leq\frac{b-a}{4} \biggl[ \frac{q-1}{\alpha(q-r)+q-1} \biggr] ^{1-{\frac{1}{q}}} \biggl[ 2\biggl(\frac{1}{s+ \alpha r+1}\biggr)^{\frac{1}{q}} \biggl\vert f'\biggl(\frac{a+b}{2}\biggr) \biggr\vert \\ &\quad\quad{} +m^{\frac{1}{q}}B( \alpha r+1,s+1)^{\frac{1}{q}} \biggl( \biggl\vert f'\biggl( \frac{a}{m}\biggr) \biggr\vert + \biggl\vert f'\biggl( \frac{b}{m}\biggr) \biggr\vert \biggr) \biggr] , \end{aligned} $$

*where* $B(x,y)$ *is the classical beta function*.

### Proof

Since $\vert f'\vert ^{q}$ is $(s,m)$-convex in the second sense on $[a,b]$ for $(s,m)\in(0,1]^{2}$, one can get the following inequalities:

2.15 $$\begin{aligned}& \begin{aligned}[b] & \int_{0}^{1}t^{\alpha} \biggl\vert f'\biggl(t{\frac{a+b}{2}}+(1-t)a\biggr) \biggr\vert \,dt \\ &\quad \leq\biggl( \int_{0}^{1}t^{\frac{\alpha(q-r)}{q-1}}\,dt\biggr)^{1-{\frac{1}{q}}} \biggl( \int_{0}^{1}t^{\alpha r} \biggl\vert f'\biggl(t{\frac{a+b}{2}}+(1-t)a\biggr) \biggr\vert ^{q}\,dt\biggr)^{ \frac{1}{q}} \\ &\quad \leq\biggl( \int_{0}^{1}t^{\frac{\alpha(q-r)}{q-1}}\,dt\biggr)^{1-{\frac{1}{q}}} \biggl[ \int_{0}^{1} t^{s+\alpha r} \biggl\vert f'\biggl(\frac{a+b}{2}\biggr) \biggr\vert ^{q}\,dt + \int_{0} ^{1}mt^{\alpha r}(1-t)^{s} \biggl\vert f'\biggl(\frac{a}{m}\biggr) \biggr\vert ^{q}\,dt \biggr] ^{\frac{1}{q}}\hspace{-20pt} \\ &\quad = \biggl[ \frac{q-1}{\alpha(q-r)+q-1} \biggr] ^{1-{\frac{1}{q}}} \biggl[ \frac{1}{s+ \alpha r+1} \biggl\vert f'\biggl(\frac{a+b}{2}\biggr) \biggr\vert ^{q} \\ &\quad\quad{} +mB(\alpha r+1,s+1) \biggl\vert f' \biggl(\frac {a}{m}\biggr) \biggr\vert ^{q} \biggr] ^{\frac{1}{q}}, \end{aligned} \end{aligned}$$

2.16 $$\begin{aligned}& \begin{aligned}[b] & \int_{0}^{1}t^{\alpha} \biggl\vert f'\biggl(t{\frac{a+b}{2}}+(1-t)b\biggr) \biggr\vert \,dt \\ &\quad \leq \biggl[ \frac{q-1}{\alpha(q-r)+q-1} \biggr] ^{1-{\frac{1}{q}}} \biggl[ \frac{1}{s+\alpha r+1} \biggl\vert f'\biggl(\frac{a+b}{2}\biggr) \biggr\vert ^{q} \\ &\quad\quad{} +mB(\alpha r+1,s+1) \biggl\vert f' \biggl( \frac{b}{m}\biggr) \biggr\vert ^{q} \biggr] ^{\frac{1}{q}}. \end{aligned} \end{aligned}$$

Therefore, inequalities (2.6), (2.15), (2.16) and the following relation imply (2.14)

$$\sum_{i=1}^{n}(a_{i}+b_{i})^{l} \leq\sum_{i=1}^{n}{a_{i}}^{l}+ \sum_{i=1}^{n}{b_{i}}^{l} $$

for $0< l<1$, $a_{1},a_{2},\ldots,a_{n}\geq0 $, $b_{1},b_{2},\ldots,b _{n}\geq0 $. □

### Corollary 2.3

*Suppose that all the assumptions of Theorem *2.3 *are satisfied*, *and if* $r=0$, *then the following inequality for fractional integrals holds*:

2.17 $$ \begin{aligned}[b] & \biggl\vert f\biggl(\frac{a+b}{2}\biggr)+ \frac{\Gamma(\alpha+1)}{b-a} \biggl[ \biggl( \frac{2}{a-b}\biggr)^{\alpha-1}J_{{(\frac{a+b}{2})}^{+}}^{\alpha}f(a)- \biggl( \frac{2}{b-a}\biggr)^{\alpha-1}J_{{(\frac{a+b}{2})}^{+}}^{\alpha}f(b) \biggr] \biggr\vert \\ &\quad \leq\frac{b-a}{4} \biggl(\frac{q-1}{\alpha q+q-1}\biggr)^{\frac{q-1}{q}} \biggl( \frac{1}{s+1}\biggr)^{\frac{1}{q}} \\ &\quad\quad{}\times \biggl[ 2 \biggl\vert f'\biggl(\frac{a+b}{2}\biggr) \biggr\vert +m^{\frac{1}{q}}\biggl( \biggl\vert f'\biggl( \frac{a}{m}\biggr) \biggr\vert + \biggl\vert f'\biggl(\frac{b}{m}\biggr) \biggr\vert \biggr) \biggr] . \end{aligned} $$

### Corollary 2.4

*Suppose that all the assumptions of Theorem *2.3 *are satisfied*, *and if* $r=0$, $\alpha=1$, *then we have the following inequality which was obtained in* [5]:

2.18 $$ \begin{aligned}[b] & \biggl\vert f \biggl( \frac{a+b}{2} \biggr) -\frac{1}{b-a} \int _{a}^{b}f(t)\,dt \biggr\vert \\ &\quad \leq\biggl(\frac{b-a}{4}\biggr) \biggl(\frac{q-1}{2q-1} \biggr)^{\frac{q-1}{q}} \biggl(\frac{1}{s+1}\biggr)^{ \frac{1}{q}} \\ &\quad\quad{}\times \biggl[ 2 \biggl\vert f'\biggl(\frac{a+b}{2}\biggr) \biggr\vert +m^{\frac{1}{q}}\biggl( \biggl\vert f'\biggl( \frac{a}{m}\biggr) \biggr\vert + \biggl\vert f'\biggl(\frac{b}{m}\biggr) \biggr\vert \biggr)\biggr] . \end{aligned} $$

### Corollary 2.5

*Suppose that all the assumptions of Theorem *2.3 *are satisfied*, *and if we let* $r=1$, *then the following inequality for fractional integrals holds*:

2.19 $$ \begin{aligned}[b] & \biggl\vert f\biggl(\frac{a+b}{2}\biggr)+ \frac{\Gamma(\alpha+1)}{b-a} \biggl[ \biggl( \frac{2}{a-b}\biggr)^{\alpha-1}J_{{(\frac{a+b}{2})}^{+}}^{\alpha}f(a)- \biggl( \frac{2}{b-a}\biggr)^{\alpha-1}J_{{(\frac{a+b}{2})}^{+}}^{\alpha}f(b) \biggr] \biggr\vert \\ &\quad \leq\frac{b-a}{4} \biggl[\frac{1}{\alpha+1}\biggr]^{1-{\frac{1}{q}}} \biggl[2 \biggl( \frac{1}{s+ \alpha+1} \biggr) ^{\frac{1}{q}} \biggl\vert f'\biggl(\frac{a+b}{2}\biggr) \biggr\vert \\ &\quad\quad{} +m^{\frac{1}{q}}B( \alpha+1,s+1)^{\frac{1}{q}}\biggl( \biggl\vert f'\biggl( \frac{a}{m}\biggr) \biggr\vert + \biggl\vert f'\biggl( \frac{b}{m}\biggr) \biggr\vert \biggr)\biggr]. \end{aligned} $$

### Corollary 2.6

*Suppose that all the assumptions of Theorem *2.3 *are satisfied*, *and if* $r=1$, $\alpha=1$, *then we have the following inequality which is the result in* [5]:

2.20 $$ \begin{aligned}[b] & \biggl\vert f\biggl(\frac{a+b}{2}\biggr)+ \frac{\Gamma(\alpha+1)}{b-a} \biggl[ \biggl( \frac{2}{a-b}\biggr)^{\alpha-1}J_{{(\frac{a+b}{2})}^{+}}^{\alpha}f(a)- \biggl( \frac{2}{b-a}\biggr)^{\alpha-1}J_{{(\frac{a+b}{2})}^{+}}^{\alpha}f(b) \biggr] \biggr\vert \\ &\quad \leq\frac{b-a}{8}\biggl(\frac{2}{s+2}\biggr)^{\frac{1}{q}} \biggl[ 2 \biggl\vert f'\biggl( \frac{a+b}{2}\biggr) \biggr\vert +m^{\frac{1}{q}}\biggl(\frac{1}{s+1}\biggr)^{\frac{1}{q}}\biggl( \biggl\vert f'\biggl( \frac{a}{m}\biggr) \biggr\vert + \biggl\vert f'\biggl(\frac{b}{m}\biggr) \biggr\vert \biggr) \biggr] . \end{aligned} $$

### Theorem 2.4

*Let* *f* *be defined on* *I* *and differentiable on* $I^{o}$ *such that* $a,b\in I$ *with* $a< b$, $f'\in L[a,b]$. *If* $\vert f'\vert ^{q}$ *is* *s*-*concave in the second sense on* $[a,b]$ *for some fixed* $q>1$ *and* $s\in(0,1]$, *then the following inequality holds*:

2.21 $$ \begin{aligned}[b] & \biggl\vert f\biggl(\frac{a+b}{2}\biggr)+ \frac{\Gamma(\alpha+1)}{b-a} \biggl[ \biggl( \frac{2}{a-b}\biggr)^{\alpha-1}J_{{(\frac{a+b}{2})}^{+}}^{\alpha}f(a)- \biggl( \frac{2}{b-a}\biggr)^{\alpha-1}J_{{(\frac{a+b}{2})}^{+}}^{\alpha}f(b) \biggr] \biggr\vert \\ &\quad \leq\frac{b-a}{4} \biggl[ \frac{q-1}{\alpha q+q-1} \biggr] ^{\frac{1}{p}} 2^{\frac{s-1}{q}} \biggl[ \biggl\vert f'\biggl(\frac{3a+b}{4} \biggr) \biggr\vert + \biggl\vert f'\biggl(\frac{a+3b}{4} \biggr) \biggr\vert \biggr] , \end{aligned} $$

*where* $\frac{1}{p}+\frac{1}{q}=1$.

### Proof

From Lemma 2.1 and by using Hölder’s inequality for $q>1$ and $p=\frac{q}{q-1}$, it follows that

2.22 $$\begin{aligned} \begin{aligned}[b] & \biggl\vert f\biggl(\frac{a+b}{2}\biggr)+ \frac{\Gamma(\alpha+1)}{b-a} \biggl[ \biggl( \frac{2}{a-b}\biggr)^{\alpha-1}J_{{(\frac{a+b}{2})}^{+}}^{\alpha}f(a)- \biggl( \frac{2}{b-a}\biggr)^{\alpha-1}J_{{(\frac{a+b}{2})}^{+}}^{\alpha}f(b) \biggr] \biggr\vert \\ &\quad \leq\frac{b-a}{4} \biggl[ \int_{0}^{1}t^{\alpha} \biggl\vert f'\biggl(t{\frac{a+b}{2}}+(1-t)a\biggr) \biggr\vert \,dt+ \int_{0}^{1}t^{\alpha} \biggl\vert f'\biggl(t{\frac{a+b}{2}}+(1-t)b\biggr) \biggr\vert \,dt \biggr] \\ &\quad \leq\frac{b-a}{4} \biggl( \int_{0}^{1}t^{\frac{\alpha q}{q-1}}\,dt\biggr)^{ \frac{1}{p}} \biggl[ \biggl( \int_{0}^{1} \biggl\vert f'\biggl(t{ \frac{a+b}{2}}+(1-t)a\biggr) \biggr\vert ^{q}\,dt \biggr)^{ \frac{1}{q}} \\ &\quad\quad{} + \int_{0}^{1} \biggl\vert f'\biggl(t{ \frac{a+b}{2}}+(1-t)b\biggr) \biggr\vert ^{q}\,dt)^{ \frac{1}{q}} \biggr] . \end{aligned} \end{aligned}$$

Since $\vert f'\vert ^{q}$ is *s*-concave on $[a,b]$, so by using inequality (1.4), we obtain

$$\int_{0}^{1} \biggl\vert f'\biggl(t{ \frac{a+b}{2}}+(1-t)a\biggr) \biggr\vert ^{q}\,dt \leq2^{s-1} \biggl\vert f'\biggl( \frac{3a+b}{4}\biggr) \biggr\vert ^{q}. $$

Analogously,

$$\int_{0}^{1} \biggl\vert f'\biggl(t{ \frac{a+b}{2}}+(1-t)b\biggr) \biggr\vert ^{q}\,dt \leq2^{s-1} \biggl\vert f'\biggl( \frac{a+3b}{4}\biggr) \biggr\vert ^{q}. $$

Using the last two inequalities in (2.22), we get (2.21). This completes the proof of the theorem. □

### Corollary 2.7

*Suppose all the assumptions of Theorem *2.4 *are satisfied*, *and assume that* $\vert f'\vert $ *is a linear map*, *then we get the following inequality*:

2.23 $$ \begin{aligned}[b] & \biggl\vert f\biggl(\frac{a+b}{2}\biggr)+ \frac{\Gamma(\alpha+1)}{b-a} \biggl[ \biggl( \frac{2}{a-b}\biggr)^{\alpha-1}J_{{(\frac{a+b}{2})}^{+}}^{\alpha}f(a)- \biggl( \frac{2}{b-a}\biggr)^{\alpha-1}J_{{(\frac{a+b}{2})}^{+}}^{\alpha}f(b) \biggr] \biggr\vert \\ &\quad \leq\frac{b-a}{4} \biggl[ \frac{q-1}{\alpha q+q-1} \biggr] ^{\frac{1}{p}} 2^{\frac{s-1}{q}} \bigl\vert f'(a+b) \bigr\vert . \end{aligned} $$

### Theorem 2.5

*Let* *f* *be defined on* *I* *and differentiable on* $I^{o}$ *such that* $a,b\in I$ *with* $a< b$, $f'\in L[a,b]$. *If* $\vert f'\vert ^{q}$ *is concave in the second sense on* $[a,b]$ *for some fixed* $q\geq1$ *and* $s\in(0,1]$, *then the following inequality holds*:

2.24 $$ \begin{aligned}[b] & \biggl\vert f\biggl(\frac{a+b}{2}\biggr)+ \frac{\Gamma(\alpha+1)}{b-a} \biggl[ \biggl( \frac{2}{a-b}\biggr)^{\alpha-1}J_{{(\frac{a+b}{2})}^{+}}^{\alpha}f(a)- \biggl( \frac{2}{b-a}\biggr)^{\alpha-1}J_{{(\frac{a+b}{2})}^{+}}^{\alpha}f(b) \biggr] \biggr\vert \\ &\quad \leq\frac{b-a}{4(\alpha+1)} \biggl[ \biggl\vert f'\biggl( \frac{(\alpha+3)a+(\alpha+1)b}{2( \alpha+2)}\biggr) \biggr\vert + \biggl\vert f'\biggl( \frac{(\alpha+1)a+(\alpha+3)b}{2(\alpha+2)}\biggr) \biggr\vert \biggr] . \end{aligned} $$

### Proof

By the concavity of $\vert f'\vert ^{q}$ on $[a,b]$ and the power-mean inequality, we have that $\vert f'\vert $ is also concave on $[a,b]$.

Accordingly, using Jensen’s integral inequality, we obtain

2.25 $$ \begin{aligned}[b] & \int_{0}^{1}t^{\alpha} \biggl\vert f'\biggl(t{\frac{a+b}{2}}+(1-t)a\biggr) \biggr\vert \,dt \\ &\quad \leq\biggl( \int_{0}^{1}t^{\alpha}\,dt\biggr) \biggl\vert f'\biggl(\frac{\int_{0}^{1}t^{\alpha}(t {\frac{a+b}{2}}+(1-t)a)\,dt}{\int_{0}^{1}t^{\alpha}\,dt}\biggr) \biggr\vert \\ &\quad =\frac{1}{\alpha+1} \biggl\vert f'\biggl( \frac{(\alpha+3)a+(\alpha+1)b}{2(\alpha+2)}\biggr) \biggr\vert . \end{aligned} $$

Analogously,

2.26 $$ \begin{aligned}[b] & \int_{0}^{1}t^{\alpha} \biggl\vert f'\biggl(t{\frac{a+b}{2}}+(1-t)b\biggr) \biggr\vert \,dt \\ &\quad \leq\biggl( \int_{0}^{1}t^{\alpha}\,dt\biggr) \biggl\vert f'\biggl(\frac{\int_{0}^{1}t^{\alpha}(t {\frac{a+b}{2}}+(1-t)b)\,dt}{\int_{0}^{1}t^{\alpha}\,dt}\biggr) \biggr\vert \\ &\quad =\frac{1}{\alpha+1} \biggl\vert f'\biggl( \frac{(\alpha+1)a+(\alpha+3)b}{2(\alpha+2)}\biggr) \biggr\vert . \end{aligned} $$

Using inequalities (2.25) and (2.26) in (2.6), we get (2.24). This completes the proof of the theorem. □

### Corollary 2.8

*Suppose that all the assumptions of Theorem *2.5 *are satisfied*, *and assume that* $\vert f'\vert $ *is a linear map*, *then we get the following inequality*:

2.27 $$ \begin{aligned}[b] & \biggl\vert f\biggl(\frac{a+b}{2}\biggr)+ \frac{\Gamma(\alpha+1)}{b-a} \biggl[ \biggl( \frac{2}{a-b}\biggr)^{\alpha-1}J_{{(\frac{a+b}{2})}^{+}}^{\alpha}f(a)- \biggl( \frac{2}{b-a}\biggr)^{\alpha-1}J_{{(\frac{a+b}{2})}^{+}}^{\alpha}f(b) \biggr] \biggr\vert \\ &\quad \leq\frac{b-a}{4(\alpha+1)} \bigl\vert f'(a+b) \bigr\vert . \end{aligned} $$

### Proof

It is a direct consequence of Theorem 2.5 and using the linearity of $\vert f'\vert $. □

### Remark 3

When $\vert f'\vert ^{q}$ is concave in the second sense on $[a,b]$ for some fixed $q\geq1$ and $\vert f'\vert $ is a linear map, the error bound in (2.27) is easier to calculate as compared to calculating it in (2.24).

### Corollary 2.9

*Suppose that all the assumptions of Theorem *2.5 *are satisfied*, *and if* $\alpha=1$, *then*

2.28 $$ \begin{aligned}[b] & \biggl\vert f \biggl( \frac{a+b}{2} \biggr) -\frac{1}{b-a} \int _{a}^{b}f(t)\,dt \biggr\vert \\ &\quad \leq\biggl(\frac{b-a}{8}\biggr)\biggl[ \biggl\vert f' \biggl(\frac{2a+b}{3}\biggr) \biggr\vert + \biggl\vert f' \biggl(\frac{a+2b}{3}\biggr) \biggr\vert \biggr]. \end{aligned} $$

### Corollary 2.10

*Suppose that all the assumptions of Theorem *2.5 *are satisfied*, *and assume that* $\vert f'\vert $ *is a linear map*, *and if* $\alpha=1$, *then we get the following inequality*:

2.29 $$ \begin{aligned}[b] & \biggl\vert f \biggl( \frac{a+b}{2} \biggr) -\frac{1}{b-a} \int _{a}^{b}f(t)\,dt \biggr\vert \\ &\quad \leq\biggl(\frac{b-a}{8}\biggr) \bigl\vert f'(a+b) \bigr\vert . \end{aligned} $$

### Proof

It is from Corollary 2.8. □

### Remark 4

The error bound in (2.29) is easier to calculate as compared to calculating it in (2.28), when $\vert f'\vert ^{q}$ is concave in the second sense on $[a,b]$ for some fixed $q\geq1$ and $\vert f'\vert $ is a linear map.

## Conclusion

In this paper, we have obtained a new fractional integral identity, which played a key role in proving our main inequalities. Several Hermite–Hadamard type fractional integral inequalities presented here, being very general, are pointed out to be specialized to yield some known results.
